# Surgical treatment strategy for recurrent parastomal hernia: Experiences from 17 cases

**DOI:** 10.3389/fsurg.2022.928743

**Published:** 2022-08-02

**Authors:** Li Luan, Qiaonan Liu, Changjin Cui, Yugang Cheng, Guangyong Zhang, Bo Li

**Affiliations:** ^1^ Department of General Surgery, The First Affiliated Hospital of Shandong First Medical University (Shandong Provincial Qianfoshan Hospital), Jinan, China; ^2^ Postgraduate Department, Shandong First Medical University, Jinan, China; ^3^ Postgraduate Department, Shandong University, Jinan, China

**Keywords:** recurrent parastomal hernia, laparoscopy, parastomal hernia, recurrent, hernia

## Abstract

**Purpose:**

This study aimed to investigate the surgical treatment strategy of recurrent parastomal hernia (PH) and show its safety, feasibility, and outcomes at a mid-term follow-up.

**Methods:**

A total of 17 cases of recurrent PH treated at our hospital between January 2016 and October 2021 were included in this retrospective analysis. Patient characteristics were recorded, and the classification of PH, operative time, intraoperative blood loss, hernia repair techniques, follow-up times, complications, as well as recurrence were compared and analyzed.

**Results:**

Altogether, 17 patients with recurrent PH underwent successful hernia repair *via* surgical treatment at The First Affiliated Hospital of Shandong First Medical University and Shandong University Qilu Hospital. No recurrence or severe complications were noted during follow-ups (mean 32.8 ± 3.77 [range 3–68] months).

**Conclusions:**

For recurrent PH, selecting a suitable repair approach based on intraperitoneal conditions such as infection, abdominal adhesions, or the length of the bowel loop, can help in achieving better therapeutic results. The lap-redo + Sugarbaker technique is worth recommending when the appropriate conditions are met.

## Introduction

Parastomal hernia (PH) is defined as an “abnormal protrusion of the contents of the abdominal cavity through an abdominal wall defect created during placement of a colostomy, ileostomy, or ileal conduit stoma,” and as a high dynamic hernia, is one the most difficult types of abdominal wall hernias to repair (1). After repair surgery, intestinal motility may cause the bowel loops to return to the hernia sac, causing a recurrent PH. Previous literature has reported that more than half of all patients with PH experience postoperative complications, with a postoperative recurrence rate as high as 45% (2–4). Owing to stoma care difficulties, pain, discomfort, and intestinal obstruction, patients with recurrent PHs may experience poor quality of life (5–7).

Recurrent PHs can be classified according to the European Hernia Society (EHS) classification method, which distinguishes the size of the defect and whether it is combined with incisional hernia (8).

Surgical options include simple suture repair, prosthetic mesh repair, and stoma site relocation (9–13). There are differences between the treatment of recurrent and primary PHs. Recurrent PH treatment is especially complicated due to recurrence and may have heavy postoperative adhesions, infections, or unknown special circumstances. Repair results are often unsatisfactory with a high recurrence rate. The management of recurrent PH remains a challenge for surgeons, and the gold standard technique has not yet been established. With only two articles that have specifically targeted the population with recurrent PH, evidence for its surgical treatment is still lacking (14, 15).

In this study, we retrospectively analyzed 17 cases of recurrent PH and summarized the surgical treatment strategies for recurrent PH. We believe that symptomatic recurrent PH requires active surgery treatment, and the suitable repair approach needs to be selected according to the intraoperative situation. The lap-re-do + Sugarbaker technique is particularly recommended when specific conditions are met. We hypothesized that this treatment strategy for recurrent PH repair would decrease the recurrence rates and perioperative complications.

## Materials and methods

This study retrospectively analyzed the details of 17 patients who had undergone recurrent PH repair at The First Affiliated Hospital of Shandong First Medical University and Shandong University Qilu Hospital between January 2016 and October 2021. Gender, body mass index, age, comorbidities, symptoms, ostomy indication, stoma type, and classification of PH were included in the patients' data. Surgical details of all PH repairs after ostomy, including surgical date, surgical technique, and mesh type, were recorded. All patients underwent computed tomography to assess the hernia aspect and to help choose the best surgical strategy. Recurrent PHs were classified according to the EHS Classification for Parastomal Hernia. For 17 cases, we used diverse surgical procedures and demonstrated the feasibility of our treatment strategy for recurrent PH by assessing the intraoperative and postoperative recovery and follow-up of the long-term therapeutic effect. Intraoperative and post-operative complications including bleeding, subcutaneous emphysema, surgical site infection (SSI), pain, intestinal obstruction, seroma formation, 30-day readmission, as well as recurrence were compared and analyzed. For this retrospective study, the hospital's ethics committee waived the need for informed consent.

For asymptomatic recurrent PHs, conservative treatment such as weight loss, abdominal support belt, health education, and regular review may be performed. For symptomatic recurrent PHs, which seriously affect the lives of patients, surgical treatment is required. The indications for surgery are as follows: inability to return the contents of the hernia sac to the abdominal cavity, the possibility of intestinal incarceration and necrosis, stomal prolapse causing incomplete or complete bowel obstruction or poor stomal function, and protuberant hernia sac causing colostomy bag leakage which affects the patient's ostomy care and normal daily activities.

Each patient completed preoperative examination, including cardiopulmonary function and coagulation test, to exclude a contraindication for surgery. Appropriate antibiotics were administered according to medication indications during the perioperative period. Preoperative bowel preparations were performed routinely. All patients underwent venous thromboembolism evaluation and prevention of deep venous thrombosis of the lower extremity.

### Surgical procedures

**Figure 1:**
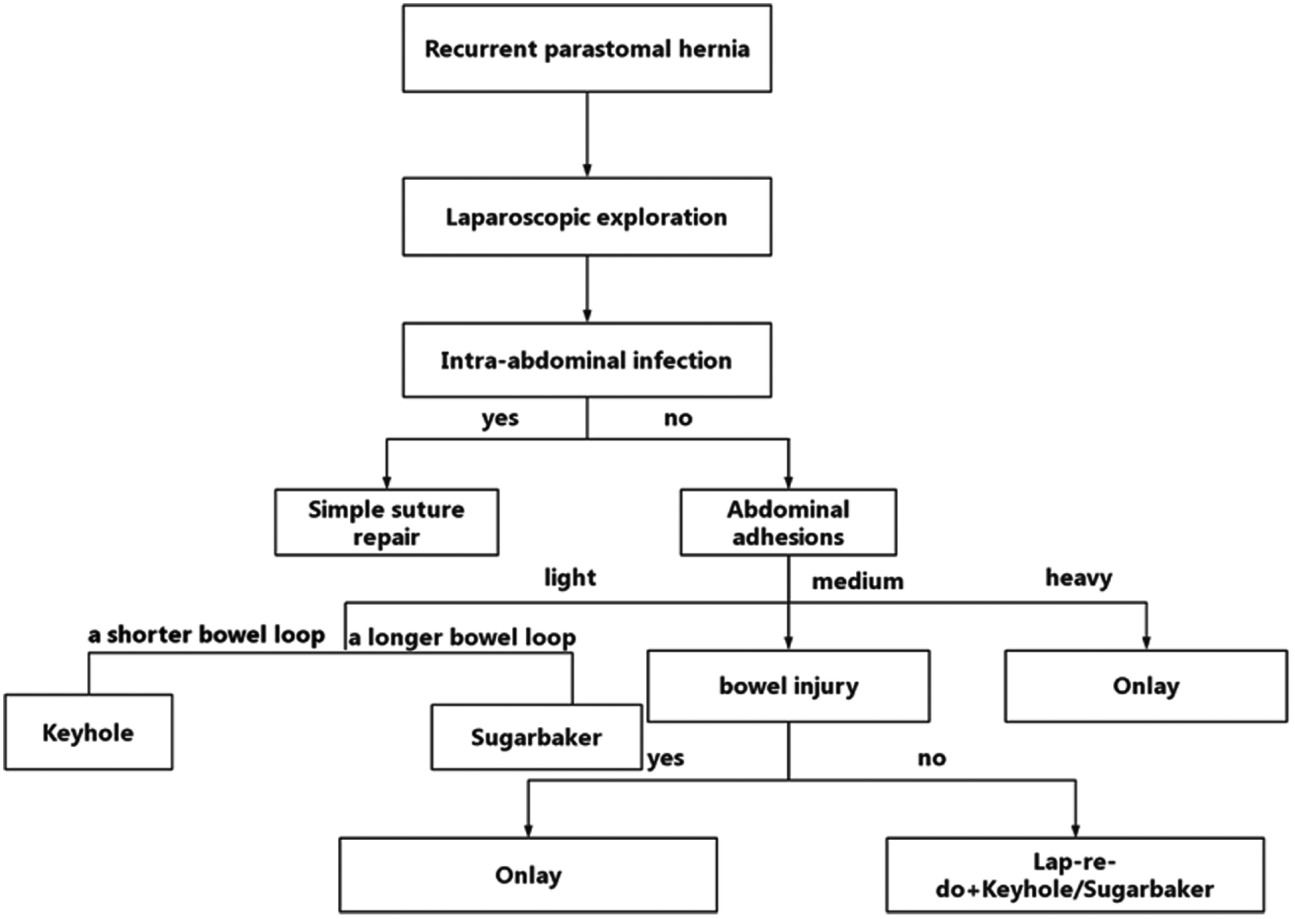
Flowchart of the surgical strategy.

**Figure 2:**
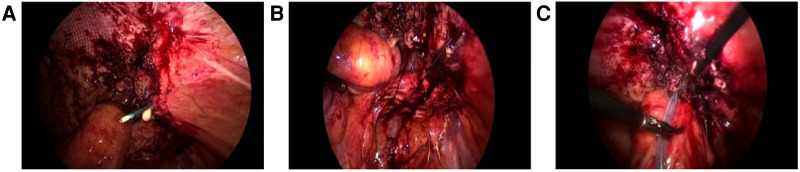
Simple suture repair (**A**) Discontinuous suture of hernia ring with hook needle, pus can be seen at the puncture; (**B**) Complete closure of the hernia ring; (**C**) Placement of drainage tube.

The surgical strategy is summarized in the following flowchart (Figure 1). After successful general anesthesia and routine catheterization, abdominal exploration was performed. Laparoscopy was performed to observe for potential infection, adhesions, tumor recurrence, or metastasis. If there was a sign of infection in the abdominal cavity, simple suture repair surgery was performed without mesh placement (Figure 2).

**Figure 3:**
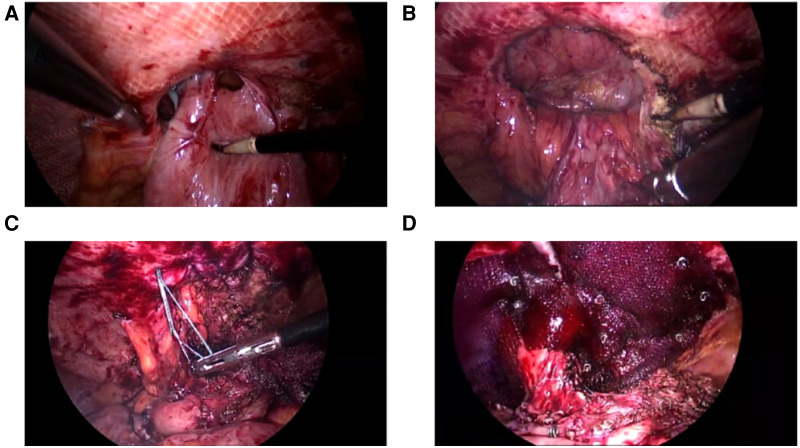
Keyhole technique (**A**) Reduction of the hernia sac contents; (**B**) The hernia ring is completely exposed; (**C**) Complete closure of the hernia ring; (**D**) Mesh fixation.

**Figure 4:**
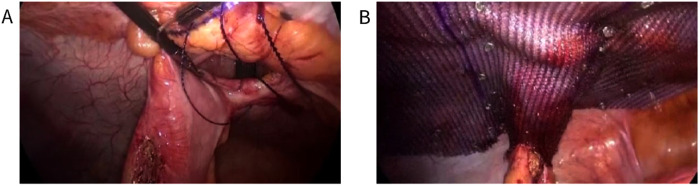
Sugarbaker technique (**A**) Complete closure of the hernia ring by continuous suture of fishbone thread; (**B**) Intraoperative view of the fixed mesh and colostomy.

Adhesiolysis was performed next, and adhesion severity was assessed in the meantime. The abdominal wall is required to be fully exposed for at least 10 cm and surrounding the hernia ring such that the mesh may be adequately placed. If the adhesion was light, the bowel loops may be safely separated *via* the laparoscopy. The length of the stoma bowel loop and the angle between the abdominal wall and bowel loop were observed to decide whether the Keyhole technique or Sugarbaker technique may be selected. The Keyhole technique is recommended for cases with a larger angle (angle of the abdominal wall >45°) and a shorter bowel loop (Figure 3), whereas the Sugarbaker technique is recommended for cases with a smaller angle (angle of the abdominal wall <20°) and longer bowel loop (Figure 4). The bowel loop may be pushed to the lateral abdominal wall such that the mesh could cover it. The central hole of the mesh should neither be too loose to cause recurrence nor too tight, in order to prevent obstructive symptoms in the Keyhole technique. In the Sugarbaker technique, precise fixation of mesh unto the surrounding abdominal wall fascia is important to prevent recurrence. The mesh should be made to cover the intestinal tube for at least 7 cm using a non absorbable nail gun. The hernia ring is closed using a non-absorbable suture. In this study, the Sugarbaker technique was preferred when these conditions were met.

**Figure 5:**
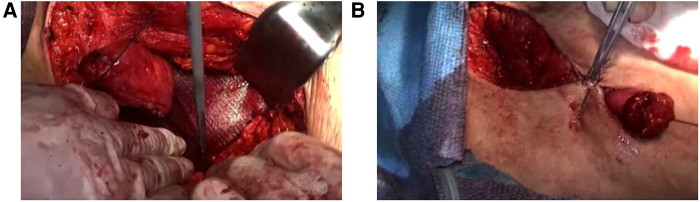
Onlay (**A**) The mesh is placed anterior to the anterior rectus sheath; (**B**) Closure of the the incision.

Next, we assessed whether bowel injuries occurred during adhesiolysis. When we found that the bowel loop may be damaged in the process of adhesiolysis, onlay was performed to avoid intestinal fistula and infection (Figure 5).

**Figure 6:**
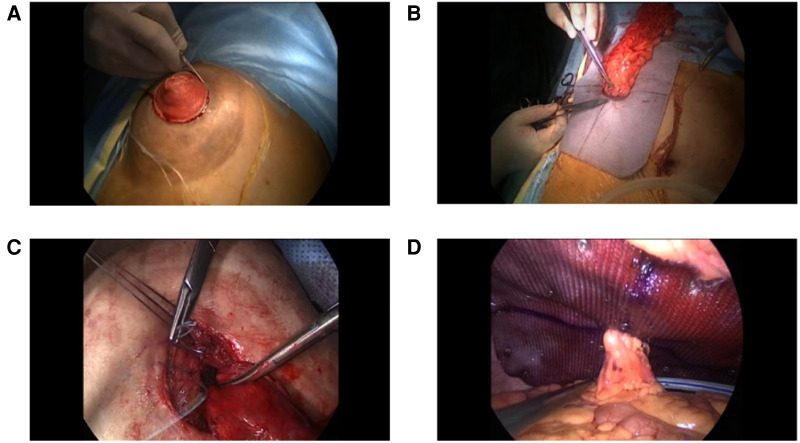
lap-redo + Keyhole (**A**) In situ incision; (**B**) Redundant stomal bowel is pulled out from the abdominal cavity, and the mesh is stitched to the sero muscular layer of the stomal tube; (**C**) Closure of the hernia ring under direct vision; (**D**) Intraoperative view of the fixed mesh and colostomy.

If bowel injury was not suspected, the lap-redo + Sugarbaker or lap-redo + Keyhole was performed under direct vision, with an adequate operating space to dissect and separate the adhered bowel and close the hernia ring tightly (Figure 6). The superfluous bowel resected *via* this incision may reduce the hernia sac contents effectively. In addition, to avoid injuries to the bowel loop, the suspicious bowel loop was pulled out from the abdominal cavity *via* this incision for inspection.

Laparoscopic exploration was converted to the onlay technique when the adhesion was too extensive for the identification of anatomic structures.

### Postoperative follow-up

The median follow-up was 32.8 (range 3–68) months. Each patient was reviewed at 3, 6, 12, and 24 months post-operatively. The examination items included physical examination and computed tomography. No patients showed disease recurrence or severe complications.

## Results

The patient characteristics are described in Table 1. From January 2016 to October 2021, we treated 17 patients (mean age: 63 years, mean body mass index: 27.5 kg/m^2^) diagnosed with symptomatic recurrent PH. Among them, 15 underwent colostomy and 2 underwent ileostomy. Ostomy was indicated for 15 patients with colorectal and anal malignancy and 2 patients with inflammatory bowel disease (IBD). BARD 3DMAXTM mesh (BD, Franklin Lakes, New Jersey) was used. During the study period, multiple surgical techniques were used to repair the recurrent PH, as shown in Tables 2 and 3. The Lap-redo + Sugarbaker technique was the most frequently applied technique in 8 cases. One case was previously repaired using the Sugarbaker technique, and we removed the previous mesh. For majority of the cases, the previous mesh was not removed and the new mesh was implanted over it because mesh removal was difficult and risky. Dead space between the meshes was avoided. Perioperative and follow-up data of the patients are detailed in Table 4. The mean operative time was 280.80 ± 90.70 min. The mean postoperative hospital stay was 9.4 ± 3.53 days. The mean blood loss was 103.50 ± 40.81 ml. Perioperative complications included one case of surgical site infection treated by sufficient drainage and antibiotic administration. The mean follow-up time was 32.8 ± 3.77 (range 3–68) months, and no recurrence or severe complications occurred. One patient with chronic pain was cured after conservative treatment. No mesh erosions were found during follow-up examination.

**Table 1:** Characteristics of 17 cases who underwent recurrent parastomal hernia repair.

Characteristics	Value
Gender	
Male	8
Female	9
Age (years) ± SE	63 ± 2
BMI ± SE (Kg)	27.5 ± 0.50
Comorbidities	
Hypertension	8
Cardiovascular disease	6
Diabetes	4
Symptoms	
ostomy care difficulties	10
Stomal prolapse	4
Pain and discomfort	7
Intermittent bowel obstruction	1
Indication for ostomy	
Colorectal and anal malignancy	15
IBD	2
Stoma type	
Colostomy	15
Ileostomy	2
EHS classification of PH	
I	5
II	0
III	12
IV	0

**Table 2:** Recurrent surgery techniques we used.

Patient No.	Primary	1st recurrence	2nd recurrence	3rd recurrence
1	Lap-re-Do + Keyhole	onlay^a^		
2	unknown	Lap-re-Do + Sugarbaker^a^		
3	Lap-re-Do + Keyhole	Sugarbaker^a^		
4	simple suture repair	Lap-re-Do + Sugarbaker^a^		
5	Lap-re-Do + Keyhole	simple suture repair^a^		
6	unknown	onlay	Keyhole	Lap-re-Do + Sugarbaker^a^
7	Lap-re-Do + Keyhole	Sugarbaker^a^		
8	Keyhole	Sugarbaker	situ re-ostomy + patch remove^a^	
9	Keyhole	Lap-re-Do + Sugarbaker^a^		
10	simple suture repair	Lap-re-Do + Keyhole^a^		
11	Keyhole	simple suture repair^a^	Lap-re-Do + Sugarbaker^a^	
12	unknown	Keyhole^a^		
13	unknown	Keyhole	Lap-re-Do + Sugarbaker^a^	
14	simple suture repair	Lap-re-Do + Keyhole^a^		
15	simple suture repair	Lap-re-Do + Keyhole^a^		
16	Keyhole	Lap-re-Do + Sugarbaker^a^		
17	Keyhole	Lap-re-Do + Sugarbaker^a^		

^a^ Indicates the recurrent parastomy hernia repair surgery performed by us.

**Table 3:** Statistics of the number of surgical cases.

Technique	Value
Lap-re-Do+ Sugarbaker	8
Lap-re-Do+ Keyhole	3
Sugarbaker	2
Keyhole	1
Onlay	1
Simple suture repair	1
Situ re-ostomy+ patch remove	1

**Table 4:** Perioperative and follow-up time of the patients.

Parameter	Mean ± SE or *n*(%)
Mean OR time (min) ± SE	280.80 ± 90.70
Mean POS (days) ± SE	9.4 ± 3.53
Time to mobilization (h) ± SE	63.6 ± 12.0
Mean follow-up (months) ± SE	32.8 ± 3.77
Intraoperative complications	
Mean Blood loss (ml ± SE)	103.50 ± 40.81
Subcutaneous emphysema	0(0.0)
Post-operative complications	
SSI	1(5%)
Chronic pain	1(5%)
Intestinal obstruction	0(0.0)
Seroma formation	0(0.0)
30-Day readmission	0(0.0)
Recurrence	0(0.0)

## Discussion

In this study, we used diverse techniques in the treatment of recurrent PHs. The timing of the surgical treatment for recurrent PH must be chosen carefully. For the patients themselves, there are potential defect in wound healing and collagen metabolism (16, 17). Other patient-related risk factors include old age, chronic respiratory disorders, corticosteroid use, obesity, wound infection, and malnutrition (18–22). Immunocompromised patients may be at higher risk for recurrence (23–25). For asymptomatic recurrent PH, conservative treatment may be performed and the risk factors should be controlled. For symptomatic recurrent PH, surgical treatment is required to avoid pain and discomfort, intestinal obstruction, and stoma prolapse, which greatly affect the lives of patients.

Various techniques for PH repair have been reported in the literature, including simple suture repair, local mesh repair, and stoma site relocation. Mesh repair has a significantly lower recurrence rate after hernia repair (26, 27). There are also multiple laparoscopic techniques available for PH repair, such as the Keyhole, Sugarbaker, and sandwich technique (28, 29). In this study, the lap-redo technique was most frequently used. This procedure combines the advantages of laparoscopic techniques and open surgery (10, 30). However, there is no clear consensus or guideline for the optimal surgical treatment strategy for recurrent PH repairs at present.

In practice, there are individual differences in terms of infection, adhesion, length of bowel loop, hernia sac size, and status of intestinal prolapse. There exists no surgical modality applicable to all conditions. Without patch placement, simple suture repair is performed when signs of infection are found.

If the adhesion is light, the Sugarbaker or Keyhole technique may be performed for primary PH. The Keyhole technique is easier to perform than the Sugarbaker technique, and therefore, it is more popular. However, it has a higher incidence of recurrence because ideal closure of the hernia ring is difficult to achieve through total laparoscopy, and the central hole of the mesh is expanding over time (28, 29). Due to increased abdominal wall tension with the mesh and adhesions, the hernia ring is more difficult to close in cases with recurrent PH. When the abdominal pressure increases, the loops re-enter the hernia sac, resulting in a high recurrence rate. The Sugarbaker technique is preferred over the Keyhole technique because it reduces the risk of recurrence (10).

In cases of redundant stomal bowel in the hernial sac and large hernia sac, the lap-redo technique is needed to free the hernia contents and close the hernial ring. Since the adhesion at the hernia ring is heavier, the lap-redo technique can be performed to separate the hernia sac under direct vision and rebuild the stoma, especially for cases with intestinal prolapse. This technique has a satisfactory therapeutic effect on patients with poor stoma function and outlet obstructive constipation. Moreover, when the intestinal loop injury is suspicious or has been damaged, this incision can be used to complete intestinal resection and intestinal anastomosis. The lap-redo + Sugarbaker technique is particularly recommended when the ostomy tube is long enough. In lap-redo surgery, attention should be paid to the prevention of mesenteric vessels injury and ostomy infection.

However, recurrent PH usually causes extensive adhesion, especially in patients with multiple recurrences. Upon dissection, the adhered bowel loop and its mesenteric vessels are very easy to damage. Selecting the onlay technique in a timely manner can achieve the purpose of repair and avoid damage.

In summary, we presented recurrent PH cases matched with suitable surgical technique, with achievement of few postoperative complications, a low recurrence rate, and favorable therapeutic results at mid-term follow-up. Although the surgical treatment strategies presented herein appear promising, research using a larger sample size and longer follow-up period are needed to guarantee more promising results of this treatment strategy.

## Conclusion

For recurrent PHs with symptoms, selecting suitable repair approach based on intraperitoneal conditions such as infection, abdominal adhesions, and length of the bowel loop can achieve favorable therapeutic results. Additionally, the lap-redo + Sugarbaker technique is recommended when the required conditions are met. This our preferred approach is adequate for management; however, it may not be ideal enough and thus requires further research in the future.

## Data Availability

The original contributions presented in the study are included in the article/Supplementary Material, further inquiries can be directed to the corresponding author/s.
